# A narrative inquiry of men’s perceptions of support and masculinity: semi-structured interviews of men living with non-cancer-related lymphoedema in the United Kingdom

**DOI:** 10.1177/17449871251347841

**Published:** 2025-08-15

**Authors:** Garry Cooper, Nicola Gale, Manbinder Sidhu, Kerry Allen

**Affiliations:** Alumni, University of Birmingham, United Kingdom; Associate Dean, University of Central Lancashire, Preston, United Kingdom; Professor/Head of School, University of Birmingham, United Kingdom; Associate Professor, University of Birmingham, United Kingdom; Professor, University of Birmingham, United Kingdom

**Keywords:** disruption, lymphoedema, masculinity, narrative, support

## Abstract

**Background:**

Lymphoedema, a chronic and visible condition, can significantly impact men’s perception of support and masculinity. The absence of research in this area, along with increased interest in men’s health, led to this study, which explores men’s perceptions of support and their sense of masculinity while living with lymphoedema.

**Method:**

This qualitative study used narrative inquiry with 12 participants through online semi-structured interviews (45–90 minutes) via Zoom. Data were analysed using linguistic narrative analysis, focusing on hegemonic masculinity and the Health, Illness, Men and Masculinities (HIMM) framework.

**Results:**

The analysis revealed three main themes. All men perceived disruption following their initial diagnosis of lymphoedema, exacerbated by limited support, such as peer support groups and timely information. They then adapted and managed changes in their lives and masculinity using available behaviours and resources, such as gaining expertise and depending on partners. Their perception of masculinity evolved, incorporating their past masculine baseline with present and future expectations.

**Discussion:**

Findings suggest that hegemonic masculinity persists amid multiple masculinities disrupted by chronic conditions like lymphoedema. Some men uphold traditional masculinity, whereas others adapt their expressions of masculinity. Recognising lymphoedema’s impact on health and masculinity could inform more tailored approaches in services, policies and strategies.

## Introduction

Men in the United Kingdom (UK) and globally face significant health inequalities in both physical and mental health, with shorter life expectancies and lower help-seeking behaviours for mental and physical health issues compared to women (Global Action on Men’s Health 2021; Global Action on Men’s Health, 2020; McKenzie et al., 2018; Naylor, 2012; ONS, 2022a, 2002b;WHO, 2018). Efforts by the UK government and third sectors aim to address these disparities (Robertson and Baker, 2017), which are often linked to societal structures such as gender, class, and ethnicity (Bosson et al., 2021; Valved et al., 2021). The precarious manhood theory suggests that manhood must be continuously earned and can be easily lost, leading to compensatory behaviours that may increase health risks, such as reluctance to seek healthcare, overworking and stress (Bosson et al., 2021; Vandella and Bosson, 2013). This theory helps to offer possible explanations as to why men are at higher risks of non-communicable diseases and social isolation compared to women (Global Action on Men’s Health, 2020; McKenzie et al., 2018; ONS, 2022a). These health inequalities are of particular relevance to nursing, as nurses working in community, wound care, and district nursing services are often central to identifying and supporting men with long-term physical conditions.

A renewed focus on men’s health addresses the ‘crisis of masculinity’, with calls for a shift from this negative framing (Katarzyna, 2021; Tosh, 2005). Inspired by strategies in Brazil and Australia, the UK Women’s Equalities Committee (2018) called for an inquiry into men’s health (Global Action on Men’s Health, 2020). The UK government has since launched an inquiry to improve health equity and reduce social isolation with calls for a men’s health ambassador (UK Parliament, 2023, 2024). This initiative acknowledges past criticisms of gender-insensitive services and emphasises targeted strategies and research in men’s health (Liddon and Barry, 2021; Robertson and Baker, 2017; UK Parliament, 2023).

One area that remains underexplored in both policy and practice is the gendered experience of lymphoedema, particularly among men with non-cancer-related causes. Criticisms exist regarding support for men with and without cancer-related lymphoedema (Cooper-Stanton et al., 2022; Scerri et al., 2024). While both groups share body image concerns, their support differs: men with cancer-related lymphoedema have regular contact with healthcare professionals and peers, whereas those with non-cancer-related lymphoedema often face limited interaction over time (Cooper-Stanton et al., 2022; Scerri et al., 2024). Lymphoedema affects both sexes; further details on prevalence and causes are in Table 1.

**Table 1.** Key information on the prevalence and cause of lymphoedema in the UK.

Lymphoedema prevalence	Lymphoedema causes
**Globally**: 250 million people **General UK Population (Age)**: 3.99 per 1000 <65 years 28.76 per 1000 >84 years **Men**: 0.47–2.48 per 1000 **Women:** 2.15–5.37 per 1000	**Primary Lymphoedema** – Genetic or syndrome based resulting in abnormal lymphatic development, examples include Milroy’s disease and Turner’s syndrome.
**Lymphoedema Service Caseload (Gender):** Men 15%–22% Women 75%–82% **Lymphoedema Service Caseload (Cause)**: Primary Lymphoedema 17.5%, Secondary Lymphoedema 82.5%	**Secondary Lymphoedema** – Represents damage to the lymphatic system, examples include venous disease, trauma, obesity and cancer.

*Source*: Fletcher et al. (2024), Keeley et al. (2019), Moffatt et al. (2017), Cooper and Bagnall (2016), Rankin (2016), ILF (2006).

Lymphoedema requires self-management by both men and women, involving practices like compression therapy, skin care, exercise and lymphatic drainage (Fletcher et al., 2024; ILF, 2006; O’Donnell et al., 2020). LIMPRINT studies suggest that up to 3–5% of the UK population may be living with chronic oedema, yet many individuals remain undiagnosed or unsupported (Keeley et al., 2019; Quéré et al., 2019). Nurses, particularly in primary and community care settings, play a crucial role in recognising early symptoms, coordinating care, and supporting self-management strategies such as compression, exercise, and skin care.

Although lymphoedema affects both sexes, women are reported to cope better and have a higher quality of life than men (Scerri et al., 2024). This study aimed to explore men’s perceptions, and how these shape their behaviours and their concept of masculinity (Cooper-Stanton et al., 2022).

To investigate the impact on sense of masculinity for men with lymphoedema, a systematic review was conducted. Most studies focused on cancer-related lymphoedema, with few addressing men specifically, and none exploring masculinity theory or alignment with gender norms (Cooper-Stanton et al., 2022). The review revealed that men’s journey involved creating a ‘New Norm’, affecting their ‘Personhood’ and strategies to manage vulnerability, such as concealment (Cooper-Stanton et al., 2022: 708). These findings align with Scerri et al.’s (2024) study on cancer-related lymphoedema in men, which described the profound impact on their social lives and masculinity. However, masculinity theory and its evolution over time were not explored in that study.

The absence of studies that focus on men, and more specifically consideration of masculinity, can perpetuate biases in our understanding and delivery of care to men (Liddon and Barry, 2021; Messerschmidt, 2019; Tidhar, 2018). This study addresses the gap in our understanding, by focusing solely on men, non-cancer-related causes of lymphoedema, and exploring masculinity theory in a UK context which is absent at the present time (Cooper-Stanton et al, 2022; Evans et al., 2011; Connell and Messerschmidt, 2005).

## Hegemonic masculinity and the Health, Illness, Men and Masculinities framework

This study uses the sociological conceptual frameworks of hegemonic masculinity (Connell and Messerschmidt, 2005) and the Health, Illness, Men and Masculinities (HIMM ) framework (Evans et al., 2011) to understand how men with lymphoedema navigate shifts in their masculine identity. These frameworks offer insights into the social constructs and performative aspects of masculinity, revealing how chronic illness might reshape men’s gendred self-perception (Connell and Messerschmidt, 2005; Evans et al., 2011). Hegemonic masculinity refers to an idealised and socially dominant form of masculinity that men are expected to perform through masculine acts such as strength, independence, control, and emotional restraint (Bosson et al., 2021; Galdas et al., 2023; Messerschmidt, 2019). This ‘pattern of practice’ is enacted for both the self and others and is reinforced through work, sport, and family roles, such as fatherhood (Rome 2021; Connell and Messerschmidt, 2005: 832). However, hegemonic masculinity is culturally and historically contingent, varying across time and place (Connell and Messerschmidt, 2005; Galdas et al., 2023). When men are unable to perform masculinity in socially sanctioned ways, such as through illness, their masculine status may be destablished, leading to subordination and loss (Courtenay, 2009; Valved et al., 2021). Although hegemonic masculinity is often the dominant form, other masculinities are proposed, such as inclusive or multiple masculinities which emerge in response to this dislocation (Galdas et al., 2023; Messerschmidt, 2019). To build on this, the HIMM framework provides a structured lens to explore how masculinity is negotiated in the context of health and illness. It positions masculinity as a social determinant of health that interacts with other identity factors such as age, ethnicity, and sexuality, and shifts dynamically across the life course (Evans et al., 2011). HIMM highlights how men respond to illness in ways that can reinforce, resist, or redefine masculine norms, depending on their social context and life stage. This framework is especially useful for exploring long-term conditions like lymphoedema, where adaptation over time may involve renegotiating self-image, care practices, and sources of support.

Together, these conceptual frameworks offer a gender-senstive perspective that enhances our understanding of how men percieve and make sense of lymphoedema, not just physically, but pyschologically, and relationally. They informed the development of the following research questions:

1. What are men’s perceptions of support within their non-cancer-related lymphoedema?
2. What are men’s perceptions of masculinity within their non-cancer-related lymphoedema?

## Methodology

### Study design

Online semi-structured interviews within a qualitative narrative inquiry framework facilitated men sharing their lymphoedema experiences and the contextualisation of their narratives within their masculine identities (Caine et al. 2013; Parahoo, 2014). This approach allowed researchers to understand the impact of lymphoedema on socially constructed identities and gender roles (Boulton, 2017; Green 2013; Bury, 1982; Connell, 1993; Courtenay, 2000, 2009).

### Recruitment and sampling

Twelve men from the UK and the Republic of Ireland were purposively sampled to represent diverse cases of non-cancer-related lymphoedema. This sample size aligns with narrative inquiry standards and prior research (Clandinin, 2012; Cooper-Stanton et al., 2022), with theoretical saturation achieved when all authors agreed on the completeness of the biographical accounts (Saunders, 2018). Recruitment was promoted through national organisations, healthcare professionals, and social media platforms like X (formerly Twitter), based on specific inclusion/exclusion criteria (see Table 2). Consent was obtained after participants reviewed the information sheet. Alternative interview methods, such as recorded phone calls, were offered if Zoom was not feasible. Demographic information on age, education, ethnicity, occupation, lymphoedema location, and diagnosis duration was collected to consider socioeconomic and intersectionality factors (see Table 3).

**Table 2.** Inclusion and exclusion criteria for participation in the study.

Inclusion criteria
(1) Over 18 years of age
(2) Identify as a man
(3) Diagnosed with primary or secondary lymphoedema (non-cancer-related)
(4) Living within the UK (all four nations) and Ireland.

*Note*. Individuals not meeting the above criteria were excluded from participation.

**Table 3.** Demographics of the 12 participants by age, ethnicity, qualification, diagnosis and duration.

Participant demographics	Number of participants
Age	30–39 (*N* = 3), 40–49 (*N* = 1), 50–59 (*N* = 6), 70–80 (*N* = 2)
Ethnicity	White – British (*N* = 10), White – Irish (*N* = 1), White – Scottish (*N* = 1)
Highest qualification	GCSE (*N* = 1), Apprenticeship (*N* = 1), A-Level (*N* = 1), Degree (*N* = 7), Masters (*N* = 2)
Diagnosis type	Primary Lymphoedema (*N* = 10), Secondary Lymphoedema (*N* = 2)
Diagnosis duration (years)	0–9 (5), 10–19 (4), 20–42 (3)

### Data collection

Twelve participants completed 45–90 minute online semi-structured interviews. The process included introductions, recording interviews and posing questions about their perceptions of access to services and masculinity with lymphoedema. Questions were developed from previous research and stakeholder input (Cooper-Stanton et al., 2022; Cosgiff, 2010; Taylor, 2005). The initial analysis of five interviews identified themes that informed subsequent interviews and narrative exploration (Vasileiou et al., 2018). Online interviews are valuable in overcoming geographical distances and gained prominence during the recent pandemic (Greenhalgh et al., 2020; Hu et al., 2020; Krouwel et al., 2019; Sullivan, 2012). Interviews were recorded and stored per the ethics application and UK data protection laws (UK Government, 2018). All interviews were transcribed and formatted for linguistic narrative analysis (see Table 4).

**Table 4.** Linguistic and reflexive analysis approaches were used in the study.

Linguistic narrative analysis	Reflexive approach
**Idea unit** – pitch indicating different focus within the text	1 – Reading for the plot and the researcher’s response to the narrative.
**Lines** – include extracts from the transcript to support the idea unit.	2 – Reading for the active ‘I’ who is telling the story
**Stanza** – Group of up to four lines about a single topic.	3 – Reading for the respondent’s relationship with family and close friends
**Strophes** – the stanzas then form into related pairs.	4 – Reading for the broader social and cultural context of the respondent.
**Parts** – the strophes form the parts that represent the story as a whole.	

### Data analysis

Linguistic narrative analysis, focusing on discourse elements within narratives, was employed to deduce meaning from participants’ stories through what is ‘actually said’ and their ‘pitch signals’ (Gee 2007; Riessman 2008: 93). This involved breaking down stories into units that convey meaning, supported by textual lines grouped into stanzas and then ‘chapters’ (Riessman 2008; Tomassini et al., 2019: 4; see Tables 4 and 5). All interviews were transcribed by the researcher for data immersion and manually transferred into an Excel sheet with assigned pseudonyms (see Table 6; Holloway and Wheeler, 2010). Reflexivity incorporated the approach offered by Mauthner and Doucet (1998, 2003; see Table 4) supported by a reflexive diary, focusing upon the decisions and inferences in the analysis. Inferences were subject to discussions with co-authors every 1–2 months, to assist in the analysis and triangulation of findings (see Table 4), with initial findings shared with participants (Parahoo 2014). Initial findings led to the identification of three overarching parts within the narratives shared by men following linguistic narrative analysis (see Table 7), that reflected the social world of men, and its disruption due to lymphoedema upon their socially constructed masculinity (Boulton 2017; Bury, 1982; Connell, 1993; Courtenay, 2000; Earthy et al., 2016).

**Table 5.** The table provides a worked example of the application of linguistic analysis in the study.

Source	Transcript examples	Idea unit with lines	Stanza	Strophe	Part
Researcher	Could you tell me a little bit about yourself and then also your experience of living with lymphedema?				
Luke	Well, um, yeah, so I’m 34 now and I work as an HGV driver. Regards to lymphedema it almost just popped up out of nowhere about two years ago, well just over two years ago now. My leg started swelling in 2020.	**01** – **(Line)** It almost just popped out of nowhere. **02 – (Line)** Well just over two years ago	**1**	**Out of nowhere**	**1 – Disruption - *‘It came from nowhere’***
Luke	I was at work, one day, and I just happened to notice my left calf was quite tense quite big just you know it’s your own body something’s not right.	**01** – **(Line)** My left calf was quite tense and quite big. **02 – (Line)** It's your own body that knows something's not right.	**2**	**It’s not right**	**1 – Disruption - *‘It came from nowhere’***

**Table 6.** The table illustrates the pseudonyms names given to participants and their ages.

Name	Age	Name	Age
Luke	34	Mike	54
Alfie	37	Will	55
Ryan	39	George	56
Harry	41	Henry	56
Chris	50	Arthur	70
Jake	54	Ben	71

**Table 7.** The table illustrates the findings of the study presented as narrative parts following linguistic analysis.

Parts	Details
**Disruption – ‘*It came from nowhere’***	Men retrace the beginning of their story in which neither themselves nor services were prepared or ready to support them.
**Journey – ‘*Trying to live normally’***	Men try to live a normal life, by adapting to their lymphoedema by acquiring knowledge through experience, or available information sources.
**Masculinity – ‘*I’ve lived with it all my life’***	Men and their masculinity were tied to the actions they undertook, to minimise its effect upon them and their perceived marginalisation

### Ethics

Ethics Approval Statement The University of Birmingham Ethics Committee granted ethical approval for this study (ERN_20-1729/August 18, 2021). We assigned pseudonyms to all participants and coded their data to ensure anonymity. The study adhered to the ethical principles outlined in the Declaration of Helsinki (World Medical Association, 2022). We managed data in accordance with the Data Protection Act and the EU General Data Protection Regulation, storing all materials on password-protected computers (Data Protection Act, 2018). Participants received no financial incentives for their involvement.

## Results

Data gathered from the completion of the semi-structured interviews led to the identification of three distinct narrative parts (see Table 7). Each part considered the disruption lymphoedema had upon the lives of men, coupled with the journey they undertook and the embedded nature of masculinity.

### Part 1: Disruption – ‘It came from nowhere’

Men recounted the beginning of their experiences when the signs and symptoms of lymphoedema first manifested and were seen as the focal point for disruption. None of the men in the study had heard of lymphoedema or expected it to happen in their lives, which is exemplified by the words shared by Chris and Harry: “It came from nowhere without warning and I had a severe bout of cellulitis, which hospitalised me for two weeks” (Chris). “I can only remember when I got it in one leg, but I can’t remember how I got it in the second one” (Harry). However, for some men, the development of lymphoedema was not obvious to them but noticed by others close to them. This signified the gradual nature of the condition for some men: “When I was 17, my mum’s friend who was a nurse noticed it” (Ryan). “I first saw symptoms of lymphedema when I was 34 due to it being noticed by a friend of mine at a party” (Will). Significance to men was around the unknown nature of what was occurring to them and how this would affect their lives. This reflects that men were not prepared for the potential changes and vulnerability this may cause in their lives, as reflected by Mike: “I didn’t know what I was dealing with and how potentially dangerous it could be”.

When men began to access services to establish the cause of their swelling, this was a long process with varying levels of support. All men accessed some form of medical support, such as the General Practitioner (GP), to establish a diagnosis, as shown in the account by Ben: “It then followed at least six months of many tests at the hospital”. Not all men perceived they could rely on healthcare professionals. Whether this related to a sense of vulnerability, men wished to gain a sense of control through acquiring knowledge. This was seen more in those men aged in their 30s, such as in the account from Luke: “I’d already done my googling . . . potentially a deep vein thrombosis. I was adamant I was going to get a referral to the vascular team”. When a diagnosis was reached it had a profound impact and even crisis for men. This was not always recognised by healthcare professionals who showed a limited level of compassion. Harry’s account illustrates receiving his diagnosis: “When I was in hospital I was told, yeah you got lymphoedema and cellulitis . . . you’ve got it for life. There’s no way of getting rid of it . . . learn to live with it.” This limited ability to adapt to the needs of men is reflective of a one-size-fits-all approach as shown by Henry: “To be honest I haven’t had any help from anybody . . . you get given a leaflet” Even when information is provided, the needs of men may exceed the ability of those supporting, with one participant stating: “It is a continuous journey trying to get the answers. Personally, I think that they could do more if they had the right training” (Ryan).

Men experience disruption in their lives from the first signs and symptoms to when they receive a diagnosis. Their experiences vary, but what is a common account is a limited preparation and awareness of the significance this may have upon men. How men accommodate the changes to their health within their lives is discussed in the next theme.

### Part 2: Journey – ‘Trying to live normally’

Men endeavoured to adapt to lymphoedema, striving to maintain a sense of normalcy. This often meant pushing themselves to continue pre-illness activities, sometimes to their detriment, as described by Ryan: “Trying to live normally as a chef. I’d get wrecked [inebriated via alcohol] . . . it gave me the mental capacity to not think about my legs.” Trying to maintain and perform previous activities, or even occupations, was a first response by men, for example Ben stated: “It took time to make things work with all that I cannot do, due to the lymphoedema. At the start it was difficult, you put one little bit on top of another then all of a sudden you’ve got a massive problem. It has taken 11 years to get here.” Trying to continue with former activities is intricately linked to who they are as a man. However, they were required to accommodate the changes in lymphoedema. One participant described the impact on his ability to walk: “I still have my regular daily walk for about an hour, but no longer than that since 2017. In previous years, I’d enjoyed driving out into the countryside and having a jolly good tramp. I no longer do lengthy walks, due to it causing my leg to become painful” (Ben) Despite some men accommodating the changes, there was a sense of loss at what they had been able to undertake before having lymphoedema. This loss fed into their perception of themselves, but also how others may perceive them, which is reflected in Mike’s story: “I don’t want to be seen as having a deficiency or a weakness. You want to come out the other side, and hope everybody’s with you.”

Coming out the other side’ showed the support men needed when dealing with lymphoedema. Those closest to men provided them with psychological support in the absence of formal services, as described by Luke and Will respectively: “I can get overworked about everything . . . thankful that I had my partner. . ., to keep me level-headed. I didn’t know what I would have done without him” “I’m very fortunate I’ve got a very supportive wife, family and three sons” However, not all men had close relationships or sought support from those closest to them. Others drew on self-reliance, contextualising their current experience of lymphoedema in relation to past events, such as previous illness, which shaped their perspective, as illustrated by Ben quote: “I have a heart problem, I’ve survived, and, typically, men will. . .” For several men having access to reliable information from experts enabled them to take control of their lives, as seen in the account shared by Mike: “to give me an A to Z of it and tailored it for me”.

Men undertake journeys that vary from their starting point and feed into their behaviours and responses aligned to their perception of what is to be a man. For example, to seek support from others or to rely on themselves. In both perceptions, men needed to acquire knowledge and experience to engage in the management of lymphoedema, before adapting. Adapting led some men to perceive it as a loss from their original concept of being a man. How their masculinity was affected by lymphoedema will be explored in the next part.

### Part 3: Masculinity – ‘I’ve lived with it all my life’

Previous parts indicate how men accommodate changes in their lives through developing lymphoedema that affect their ability to engage in certain activities. Whilst these are important considerations, men’s perceptions of masculinity were also challenged by lymphoedema. Many had not reflected on their masculinity until prompted, as Arthur noted: “My masculine image is one I can’t quite articulate because I’ve lived with it all my life”. Even when men expressed their masculinity, it was rooted in the physical changes that had occurred to them, and their expected roles. As seen within the following accounts by Chris and Harry: “My masculinity at first was affected when I had to put these stockings on every day. It was incredibly embarrassing putting a stocking on in front of my wife” (Chris). “In terms of masculinity some men are like, why is that dude wearing long pants, he must be fucking baking, it’s probably 90% in my head. I don’t even like going to the gym and I used to love my legs days. I don’t even wear shorts at home when I walk by a mirror I just freak out” (Harry).

Some men acknowledged the impact this had upon them. Others felt that the condition had either no effect or was harder for women compared to men, due to the aesthetic effects. The quote by Alfie illustrates his changing perception of himself: “I don’t think it affects me in a cosmetic aspect. I think it’s probably a harder condition for women, men can disguise the issue. I’m able to wear long pants everywhere, so nobody says anything about my leg. If I got it 10 years earlier, it probably would have mentally affected me more” (Alfie). Alfie’s quote indicates how men may change their perception of their masculinity. Equally, by comparing to others, such as women, men are using certain traits, such as stoicism to support themselves by minimising the perceived impact of the condition upon their masculinity. However, for some men, there is a need to hide their condition from others, as seen in the following quote from Chris: “On holidays when we go swimming, I would be covering up my leg with a towel. I would also wear baggy trousers rather than shorts. Certain activities were limited because I feared breaking the skin and getting an infection” (Chris). Despite men’s concerns surrounding their condition, most men did not share the presence of their condition beyond partners and close friends, as seen in the following quote also by Chris: “My wife, bless her, said, you know it’s just a case of getting used to the new normal. Saying those words have stuck with me ever since. The pre-lymphoedema version of me was confident, social, healthy, and a fun guy, who took an absolute battering for the first two or three years”.

Whilst men engaged with informal support, formal places were virtually absent for men, specifically to discuss their experiences, which perpetuates a sense of marginalisation. Even when formal spaces are provided, they are not wholly suitable, as seen in the quote by Henry: “I was invited to a coffee morning support group. It was all women talking about breast cancer. They seem to think it’s a woman’s only problem,but it is more ignorance that men get lymphedema”. The absence of support groups for men can not only perpetuate the invisibility of their needs but also reinforce the need for them to rely upon themselves. Despite acknowledging the impact lymphoedema has had on their lives and sense of masculinity, the findings show that masculinity, though often intangible, remains a pervasive social influence, shaping how men percieve their experiences and navigate illness.

## Discussion

This study is among the first to explore men’s perceptions of support and the impact of lymphoedema on their sense of masculinity (Scerri, et al, 2024; Cooper-Stanton, et al, 2022). The interviews revealed three temporal dimensions within particpants’ collective narratives: initial disruption, subsequent accommodation, and long-term adaptation. In this section, the discussion examines the findings through the theoretical lenses previously outlined, hegemonic and the HIMM framework, and in relation to the broader literature on masculinity and men’s experiences of living with long term conditions.

### Disruption: ‘It came from nowhere’

Men perceived lymphoedema as a disruption in their lives, often noticed gradually or following complications. While the physical manifestation of lymphoedema is expected (ILF, 2006), the significance of lymphoedema as a ‘life event’ within the HIMM framework is unclear (Evans et al., 2011), due to a limited definition of what this may encompass. Men often embark on a ‘journey into the unknown’ seeking answers about lymphoedema’s origin and management (Cooper-Stanton et al., 2022: 22). McGarvey et al.’s (2014) study reflects the limited time men had to prepare for a long-term condition and the disruption it causes in their lives. Disruption, though not explicitly mentioned in hegemonic masculinity (Connell and Messerschmidt, 2005) or the HIMM framework (Evans, et al, 2011), affects men’s physical health and masculinity expression (Connell and Messerschmidt, 2005). The HIMM framework references disruption implicitly when men ‘define’ and ‘redefine’ their masculinity against gender norms (Evans et al., 2011: 10). Whilst hegemonic masculinity emphasises traits like independence and physical strength (Galdas et al., 2023; Connell & Messerschmidt, 2005) lymphoedema affects how men express masculinity both physically, by limiting their abilities, and socially ’, by altering their participation in activities that showcase masculinity (Connell & Messerschmidt, 2005). Alternatively, lymphoedema can represent a focal point in terms of ‘biographical disruption’ offered by Bury (1982: 167) in which progressive or sudden events affect a person’s life with a focus upon chronic illness, thus defining what a ‘life event’ in the HIMM framework may encompass (Evans et al, 2011: 10).

Men’s pursuit of answers by seeking healthcare professionals’ input reflects a response to disruptions and vulnerability, which may be perceived as being a ‘feminine’ trait (Courtenay, 2000: 1389), which is contrary to proposals in hegemonic masculinity in which men may not seek help (Connell and Messerschmidt, 2005). However, it may also represent gender norms of seeking control to reinforce and demonstrate their masculinity (Galdas et al., 2023; Messerschmidt, 2019).

Despite delays in diagnosis and care, men actively sought understanding and to manage perceived vulnerability through information-seeking, which could act as a ‘turning point’ in their lives (Bogan et al., 2007: 219). Delays in obtaining a diagnosis and the need for multiple tests are common in lymphoedema, leading to increased anxiety and complications, such as infections and even a self-perceived ‘crisis’ for the men involved (Watts and Davies, 2016; Evans et al., 2011; Perera et al., 2007; Moffatt et al., 2003). Men’s anxieties increased when there was a limited understanding of lymphoedema, perceived disinterest or lack of compassion by healthcare professionals or others, which was noted in a study by Schulze et al. (2018). This might suggest that these men are considered insubordinate to the ideals expected of men, which is reflected in actions of others around them (Connell and Messerschmidt, 2005). This is also reflective of Liddon and Barry’s (2021) suggestions of gamma bias, where pre-held notions are emphasised or deemphasised, in one or morematrixes of judgements about gender, perpetration, privilege, celebration, and victimhood. It has been suggested that limited recognition of victimhood, such as illness amongst men, or not acknowledging the positive benefits of masculinity in celebration, such as skills mastery can marginalise men in mainstream Western societies and healthcare (Seagar and Barry, 2020; Stangl et al., 2019). This is despite certain observations that these social constructs of men, such as gender norms of skills mastery, may prove useful when managing their health (Galdas et al, 2023).

Failure to recognise gender bias in care may leave men’s needs with lymphoedema unmet (Cooper-Stanton, Gale and Sidhu, 2022), as seen in studies on diabetes, arthritis, and cancer-related lymphoedema where men report loss of control, identity, and role (Flurey et al, 2018; Hamilton and Thomas, 2016; Dale etal, 2015). While hegemonic masculinity and the HIMM framework reference loss, they do not fully address the profound disruption and identity renegotiation men experience in managing long-term conditions.

### Journey: ‘Trying to live normally’

Previously, the focus was on the initial disruption men experienced; this part considers how men accommodated lymphoedema into their day to day lives. It is unclear how men adapt their gender norms and masculinity in response to life events within the hegemonic or HIMM frameworks. Beyond reference to men’s masculinity changing over ‘time’, or to moments when men ‘intentionally’ work towards a relational shift in how they engage with and percieve masculinity (Connell and Messerschmidt, 2005: 846, 853). The study by Flurey et al. (2018) offers a potential explanation, as they suggest men with chronic illness might uphold, negotiate or reject hegemonic masculinity and the gender norms associated, based on their study of men with rheumatoid arthritis. The findings of our study illustrated how men continued with past activities to assert their masculinity and potential to uphold hegemonic masculinity, which aligns with the study by Flurey et al (2018). Addittionally men in our study may have continued to persue activities to establish the boundaries that are present in their lives due to lymphoedema. This is consistent with the studies by Nixon et al, 2018 and Deng and Murphy, 2016, who focused on the impact of head and neck lymphoedema, and was noted in the systematic review by Cooper-Stanton, et al, 2022. Managing lymphoedema also allowed men to embody masculinity norms through expressing mastery of skills and knowledge, which is reflective of the performative elements of masculinity, in terms of the ‘social practice’ in hegemonic masculinity (Boulton, 2017; Connell and Messerschmidt, 2005: 851). These practices may also reflect the formation of a ‘New Norm’ suggested in the systematic review by Cooper-Stanton et al. (2022: 22), in which past activities were pursued or modified, including support networks.

Support networks were crucial, highlighting the positive outcomes of social connectedness (Arnell, 2014; McKenzie et al., 2018). Despite the potential wish for men to become experts in their condition, not all approached it the same way, reflecting multiple masculinities, such as involving partners instead of being completely self-reliant. Flurey et al. (2018) suggested that this may also demonstrate the negotiations that have or are taking place regarding hegemonic masculinity, with the incorporation of support networks as extensions in their expression of masculinity. Arnell (2014) suggests that the involvement of partners or others can be beneficial to men to improve engagement in their health and may form part of masculine capital. Masculine capital represents a resource, as a form of currency the men use, such as support networks and or knowledge acquisition, when there is a perceived deficit in the expression or the ability to fulfil gender norms surrounding their expression of masculinity, such as physical ability (Arnell, 2014). Despite masculine capital not being explicitly present in hegemonic masculinity or the HIMM framework, it offers another consideration in the way men may ‘renegotiate’ or ‘redefine’ their masculinity (Evans, 2011: 10; Flurey et al., 2018: 122).

### Masculinity: ‘I’ve lived with it all my life’

This part examines the impact of lymphoedema on men’s masculinity. Despite initial difficulties articulating their perceptions, men eventually referred to physical strength, appearance, and the loss experienced when they are unable to meet their own expectations of masculinity, such as engaging recreational activities, or appearing masculine when having to putt on compression in front of partners. This aligns to the concept of hegemonic masculinity and the vulnerability experiences when men are unable to meet social expectations (Connell and Messerschmidt, 2005) which is amplified when illness effects their perceptions of masculinity (Evans et al., 2011). Physical deterioration, while expected across a man’s life, illustrates a potential shift in gender hierarchy and status, leading to a perceived subordinate position in terms of hegemonic masculinity (Courtenay, 2009). The loss of perceived status can lead to certain behaviours, such as withdrawal from activities, or pursuing them despite risks to their health (Flurey et al., 2018). Concerns about physical appearance can be perceived as being more feminine, opposing expectations of masculinity but can also reflect societal expectations of a ‘real’ man (Courtenay, 2009: 16), in which they should appear healthy and strong (Connell and Messerschmidt, 2005).

Some men denied lymphoedema’s impact, reflecting ‘stoicism’ to avoid socially constructed’feminine’ characteristics and potential marginalisation (Evans et al., 2011: 12; Connell and Messerschmidt, 2005). Others negotiated their masculinity, leading to varied responses to accommodate lymphoedema even when this may incur perceptions of vulnerability (Cooper-Stanton et al., 2022; Flurey et al., 2018). The varied responses by men may also reflect multiple masculinities and the level of social connectedness, in which partners may be relied upon for ‘private’ emotional support, whilst other men and friends serve a more ‘instrumental’ support in terms of practical help (McKenzie et al., 2018: 1248, 1255). Access to peer support was noted as crucial for men affected by lymphoedema although this was experienced as limited (Ríos-González et al., 2021; Rios-Gonzalez et al., 2018; McKenzie, 2018; Rudman and Goodwin, 2004).

### Theory

This study deepens our understanding of how men navigate lymphoedema while maintaining socially constructed masculine ideals of control and independence (Connell and Messerschmidt, 2005; Messerschmidt, 2019). Hegemonic masculinity promotes autonomy by avoiding dependence, yet participants sought emotional support from trusted contacts, especially partners. Some men faced isolation due to their needs being unrecognised by healthcare professionals complicating their sense of masculinity. This reflects perceptions of insubordination, where deviations from traditional masculinity are undervalued, or gamma bias, which downplays men’s vulnerabilities (Liddon and Barry, 2021; Messerschmidt, 2019). Over time, shifting perceptions of masculinity revealed that men’s views on health and societal roles alter. Recognising these nuances allows for more effective engagement with men, fostering supportive environments that do not reinforce feelings of isolation. The study highlights key areas for clinicians and policymakers to enhance services (see Table 8). Implementing these practices can help address the physical and psychosocial needs of men with lymphoedema, especially regarding masculinity and identity.

**Table 8.** The table illustrates the key areas of learning for clinicians and policymakers based on the findings of the study.

Key learning points	
**Tailored Support Over Time –** The temporal elements of the lymphoedema experience suggest the need for tailored support at each stage of a man’s journey.	**Initial Disruption:** Providing early, accurate information and educational materials to help men navigate initial confusion and anxiety. **Accommodation:** Regular follow-ups and support groups to help men adapt and support men’s efforts to learn about their condition and the skills to manage it effectively. **Long-term Adaptation:** Sustaining adaptations with advanced management techniques, psychological support and specialist care.
**Addressing Masculine Identity –** The physical effects of lymphoedema on strength, appearance and physical ability are intertwined with men’s perceptions of their masculinity. Clinicians should:	**Acknowledge Concerns**: Recognising and how lymphoedema affects men’s sense of masculinity and address these concerns openly. **Facilitate Positive Redefinition**: Support men to explore and redefine their masculinity in a meaningful way. **Use the HIMM Framework:** Apply the Health, Illness and Masculinity Model to guide discussions on identity.
**Enhancing Support –** Given that men may need to redefine their masculinity in response to lymphoedema and the importance of peer support, practice should:	**Facilitate Networks:** Connect men to peer support groups to share experiences and reduce isolation. **Involve Partners:** Guide how partners can effectively support care routines. **Combat Stereotypes:** Challenge biases that minimise men’s experiences and provide care that respects their unique challenges.

### Strengths and limitations

This study explores how men perceive support for their lymphoedema and how this interacts with notions of masculinity. The focus on non-cancer-related lymphoedema is valuable due to the scarcity of literature and the application of hegemonic masculinity theory in this context. The use of narrative inquiry and linguistic narrative analysis (Gee, 1991) is a novel approach in this field, enhancing the credibility, dependability, and generalisability of the findings. The participant demographics (White men over 50) align with existing research in developed Western countries (Cooper-Stanton et al., 2022; Keeley et al., 2019; Moffatt et al., 2017). The sample size of twelve participants aligns with similar studies. While the study included participants from varied sexual orientations, the sample lacked ethnic diversity. Despite recruitment efforts across multiple platforms and organisations over 12 months, participation was limited to White men. Future research should focus on addressing this limitation through more targeted recruitment strategies aimed at engaging underrepresented ethnic groups. The transferability of results outside the UK and the Republic of Ireland may be limited due to cultural variance in constructions of masculinity; further research should consider different healthcare systems and cultural contexts. Additionally, the predominance of White, heterosexual men limits the study’s applicability to other demographic groups.

## Conclusion

The study examined men with non-cancer-related lymphoedema, focusing on their perceptions of support and masculinity. It found that men face delays in diagnosis and limited support, prompting them to rely on their resources, such as masculine capital and traits like independence to manage learning, and management of their new normal . Partners are the primary support network, providing psychological and social aid. However, the absence of male peer support can further marginalise and isolate men. Redefining their masculinity within a altered social context was a long-term process, often taking years. These findings underline the importace of gender-sensitive services that not only address men’s practical, buy also psychological needs.

**Key points for policy, practice and research**

- *Disruption of Lives*: The onset of lymphoedema came unexpectedly for many men, causing significant disruption. They experienced confusion and a lack of preparedness, particularly around diagnosis and early management. This highlights the need for policy and clinical pathways that promote earlier identification, timely diagnosis, and targeted education materials to support men at the point of diagnosis.
- *Journey of Adaptation*: Men attempted to adapt to their new reality by independently seeking information and trying to maintain a sense of normalcy. This underscores a practice gap: services should develop structured self-management support tailored to men’s learning preferences and emotional responses to chronic illness. Further research could explore how men best access, interpret, and apply condition-specific information in daily life.
- *Impact on Masculinity*: Lymphoedema had a profound effect on men’s perceptions of masculinity. Physical changes and the visible management of the condition (e.g. compression garments) often led to embarrassment and concealment, damaging self-image and confidence. Services should be informed by gender-sensitive approaches that acknowledge the psychosocial dimensions of living with chronic illness. Policy and training should support healthcare professionals to engage more effectively with male identity concerns. Future research could further examine how masculinities influence men’s help-seeking and adaptation across long-term conditions.
- *Lack of Support Structures*: The study found a significant lack of formal support structures tailored to men with lymphoedema. Existing support groups were often perceived as female-centric, contributing to feelings of marginalisation. This points to a clear need for policy and service development that funds or facilitates male-specific or mixed-gender support options, including peer-led models. Research is also needed to evaluate the impact of male peer support on wellbeing and engagement with care.
